# Supplementary material for: Prevalence and factors associated with pediatric HIV therapy failure in a tertiary hospital in Asmara, Eritrea: A 15-year retrospective cohort study
Source: PLoS One. 2023 Mar 9;18(3):e0282642. doi: 10.1371/journal.pone.0282642 (PMC9997912; doi:10.1371/journal.pone.0282642)
Supplement: Attachment — Submitted filename: Response to reviewer.docx [file pone.0282642.s002.docx]

**To PLOS ONE Editorial Office**

We would like to thank Academic Editor of PLOS ONE and our reviewer for their invaluable inputs and constructive comments that are helpful to massively improve the quality of the manuscript. After careful consideration of the points raised, the point-by-point response are as follows:

| **Minor Points raised or amendments requested by reviewer #1** | | **Authors’ Response** |
| --- | --- | --- |
| 2. | Line 52: Space between 2018 and reference 1 | The comment has been addressed |
| 3. | Line 59: Do authors mean prevalent cases instead of prevalence. | The statement has been overhauled to “*In Eritrea, a 2019 Spectrum modeling estimated that the magnitude of people living with HIV/AIDS (PLWHA) is 14, 000 (0.36%). Among these, children (<15 years) make up 4% and 8,956 (73%) patients are currently cART.*” |
| 4. | Line 95: Provide ethical approval number | Directive noted and the number is provided. |
| 5. | Line 100: The study includes children and adolescents. Please specify this. | The comment has been addressed and further description of the age breakdown is stated in the methodology section called operational definition. |
| 6. | Line 103: Is LPV/r not a recommended first line ART treatment in children in Eritrea under 3 years? | Protease inhibitors (LPV/r) are second line regimens in Eritrea as per local guidelines due the absence of syrup based formulations. So the LPV/r tablets are similarly used as second-line regimens as in adults. |
| 7. | Line 130: Please define SD | SD is defined in the beginning of the statement as “Standard deviation (SD)” |
| 8. | Line 167: Please explain why you provide a p-value without any comparison. | The comparison is now being placed and mainly compares the included vs excluded cases. |
| 9. | Table 4: For some odds ratios, you presented decimal number with one figure after the decimal others two, please  Present them the same way. | The comment has been addressed. |
| 10. | Line 384: The limitation section should be in the discussion section | Necessary changes have been undertaken. |
| 11. | Discussion may be shortened | Some sentences have been deleted. |

A. **Reviewer’s comments**

| **Major points raised or amendments requested by Reviewer #1** | | **Authors’ response** |
| --- | --- | --- |
| 1. | You excluded 12% of your cohort without any justification. Can you provide the details why they were excluded? As stated in table 1, excluded participants were mostly in advanced stage of the disease and lived outside of Maekel. The exclusion of these participants is source of selection bias which is not addressed in the manuscript. | The detailed justification for exclusion are specified in figure 1. 97 cases were excluded out of which, 54 patients were excluded due to follow-up less than 6 months.  In studies of this kind [See Costenaro P, Penazzato M, Lundin R, Rossi G, Massavon W, Patel D, et al. Predictors of Treatment Failure in HIV-Positive Children Receiving Combination Antiretroviral Therapy : Cohort Data From Mozambique and Uganda. 2015;4(1):39–48.], treatment response is evaluated at 6 months following treatment initiation. The reasoning behind this cut-off is that treatment response cannot be defined prior to this duration. An additional 42 cases were excluded due to key missing follow up data: unknown follow up duration and those with no therapy outcome endpoints. Missing data is a common problem in retrospective studies of this kind. Consequently, this statement has been in cooperated in the manuscript. |
| 2. | For the survival analysis, we suggest you include all participants and censored those lost to follow up. | This has been addressed in the methodology subsection, end-point definition as “*For TF analysis, the period of follow-up was from cART initiation up to the earliest detection of TF. Children without TF were censored at the date of death, lost to follow-up (defined as missing follow-up visits for more than 6 months), transferred to another clinic, or the date record of any last event in the clinic.*” So, yes we included all participants and censored those lost to follow up. |
| 3. | Presenting percentages in line makes it difficult to read table 1. It would be better to present the percentages in colons. | The comment has been addressed. All the percentages are across columns. |
| 4. | The study design is a retrospective study yet you report TF prevalence. In retrospective studies you cannot evaluate the  Prevalence. You also reported a cumulative incidence and incidence rate. Can the authors clarify this in the manuscript? | From epidemiologic literatures (*Biostatistics and epidemiology: a primer for health and biomedical prefessionals / by SylviaWassertheil-Smoller. —3rd ed.page 92-93*), our understanding of prevalence is:  = Number of persons with a condition / Total number of persons in population at risk for the condition at a particular point in time  In our case, our population is children living with HIV receiving cART in the study setting and the condition is therapy failure.  On the other hand, incidence means:  = Number of new cases of a disease per unit of time / Total number at risk in beginning of this time period  In our opinion, so long as patient’s information is well documented, **period** prevalence of a specific disease in a population can be calculated.  Overall, it our submission that period prevalence can be calculated using retrospective data so long as the data is well collected and the time period is specified.  The below listed references used similar approach to determine the burden (prevalence) of TF.   1. Solomon Weldegebreal Asgedom et al., Immunologic and Clinical Failure of Antiretroviral Therapy in People Living with Human Immunodeficiency Virus within Two Years of Treatment. Hindawi BioMed Research International Volume 2020, Article ID 5474103, 8 pages 2. Dow et al.: Durability of antiretroviral therapy and predictors of virologic failure among perinatally HIV-infected children in Tanzania: a four-year follow-up. BMC Infectious Diseases 2014 14:567 3. Isaac O. Abah et al: Antiretroviral Therapy-associated Adverse Drug Reactions and their Effects on Virologic Failure- A Retrospective Cohort Study in Nigeria. Current HIV Research, 2018, 16, 436-446 4. Bacha et al.: Predictors of treatment failure and time to detection and switching in HIV-infected Ethiopian children receiving first line anti-retroviral therapy. BMC Infectious Diseases 2012 12:197. |
| 5. | Adherence was assessed upon each follow-up visit based on missed doses. This variable changes with time and so this  Should be mentioned in the model. | The definition of suboptimal adherence comprises of at least one record of fair or poor adherence. Hence patients were classified as either having a record of suboptimal adherence or no record throughout their follow up period. |
| 6. | In addition, this variable was operationally defined as a categorical variable, but in  Table 4 it was instead used as a quantitative variable. | This comment has been addressed. |
| 7. | The denominator of TF is not clear from line 184 to line 187 (how did you calculate the percentages)? | Absolute numbers have been included. e.g. 279/724 for a prevalence of 38%. The denominator is total number ofCLHIV whereas the numerators utilized are number Virologic failure alone to calculate VF, Immunologic failure alone to calculate IF, clinical failure alone to calculate CF and number of patients who had virologic, immunologic and clinical failure at the same time. |
| 8. | In table 2 there is no reference for the rate ratio. | This comment has been addressed. |
| 9. | The bivariate analysis in table 3 is not appropriate. Please remove this section. | Table analyzed factors associated with the prevalence of TF. While table 2 analyses factors as per subcategories of the variables’ incidence.  However, and based on your opinion, we can either retain the table (after revising the title – e.g we have amended the title to factors associated with prevalence TF) as is generally the case in studies of this kind or attach it as a supplementary file.  We can also omit it altogether. |
| 10. | ART change during follow up is probably related to TF or vice versa, so it would not be appropriate to use this variable as a predictor. | We have substituted the word cART change with the word, cART substitution. cART substitution is not necessarily associated TF but can be linked to stock outs, adverse drug reactions and emerging of preferable regimens. cART switching is definitely due TF. Our variable merely captured cART substitution. |

B. **Journal Requirements**

| **Journal Requirements** | **Amendments made** |
| --- | --- |
| 1. Please ensure that your manuscript meets PLOS ONE's style requirements, including those for file naming. The PLOS ONE style templates can be found at | The manuscript has been formatted in line with Plos One Style. |
| 2. PLOS **only** allows data to be available upon request if there are legal or ethical restrictions on sharing data publicly.   In your revised cover letter, please address the following prompts: a) If there are ethical or legal restrictions on sharing a de-identified data set, please explain them in detail (e.g., data contain potentially sensitive information, data are owned by a third-party organization, etc.) and who has imposed them (e.g., an ethics committee). Please also provide contact information for a data access committee, ethics committee, or other institutional body to which data requests may be sent. | Data is available upon request for the following reasons:  The data The data for this paper is from routine programmatic monitoring of Eritrean Ministry of Health ART programs. As such, the data is the possession of the Ministry of Health and cannot be posted to an online repository. That said, the data can be made available on request to the Ministry of Health. Such requests would best be made through the senior author, Dr. Araia Berhane (araiabm@gmail.com) who can facilitate the request for access to the data at any time. Additionally, has a potential sensitive information as the study site is the only pediatric treatment center, there are certain identifiers that cannot be de-identified and remain analyzable. |
| 3. Please upload a new copy of Figure 3 as the detail is not clear. | Changes has been made. |
