# Supplementary material for: Prevalence and factors associated with pediatric HIV therapy failure in a tertiary hospital in Asmara, Eritrea: A 15-year retrospective cohort study
Source: PLoS One. 2023 Mar 9;18(3):e0282642. doi: 10.1371/journal.pone.0282642 (PMC9997912; doi:10.1371/journal.pone.0282642)
Supplement: Attachment — Submitted filename: Point by point response.docx [file pone.0282642.s003.docx]

**To PLOS ONE Editorial Office**

We would like to thank staff Editor of PLOS ONE and the reviewers for their invaluable inputs. Otherwise, see table below for point-by-point responses to reviewers comments and concerns.

| **Minor Points raised or amendments requested by reviewer #2** | | **Authors’ Response** |
| --- | --- | --- |
| 1. | I still see a few typos: eg, line 162, something is missing between CLHIV and the next sentence; title of table 1. Please check for similar errors. | Extensive review of the document has been undertaken to remove typos and revise problematic sentences. |
| **Points raised or amendments requested by Reviewer #3** | | **Authors’ Response** |
| 1. | (Line 28, 251, etc) The term ‘predictors’ is used in this manuscript. I believe the authors are treating this as a synonym to ‘factors’ and ‘independent variables’. As the authors didn’t build predictive model, rather they have looked in to associations. | Comment is well taken, and changes have been made. However, pardon a few comments on this issue.  First, a likelihood ratio is basically a prediction. The popularity of this term in published literature and statistical texts is meant to highlight this fact.  See the following texts for illustrations.   1. Predictors of treatment failure and time to detection and switching in HIV-infected Ethiopian children receiving first-line antiretroviral therapy. Vol. 12, BMC infectious diseases. p. 1–8. 2. Haile GS BA. Predictors of treatment failure, time to switch, and reasons for switching to second-line antiretroviral therapy in HIV-infected children receiving first-line antiretroviral therapy at a Tertiary Care Hospital in Ethiopia. BMC Pediatr. 2019;19(37). 3. Predictors of treatment failure and time to detection and switching in HIV-infected Ethiopian children receiving first-line antiretroviral therapy. Vol. 12, BMC infectious diseases. p. 1–8. 4. Costenaro P, Penazzato M, Lundin R, Rossi G, Massavon W, Patel D, et al. Predictors of Treatment Failure in HIV-Positive Children Receiving Combination Antiretroviral Therapy : Cohort Data From Mozambique and Uganda. 2015;4(1):39–48. |
| 2. | Line 123-4 Does persistent mean CD4+ levels below the threshold for six months for every measurement? | ….Less than the threshold for two consecutive measures, i.e. at baseline and the WHO recommended period of CD4+ count measurement. |
| 3. | Lines 151-156 Some of the covariates in the cox model appear to be time dependent. How were those handled? Would it be better to switch to a multiple endpoint cox-model, such as Anderson-Gill model? Also, since treatment failure probably doesn’t happen at the time of the survey, i.e., it happens between two surveys, was the outcome treated as interval censored? | Most the variables listed in the cox-model are baseline characteristics of the patients. Therefore, analysis looks at the influence of specific baseline characteristics on outcome. Exception to this is, adherence which is categorized as maladherent or good adherent due the fact that each patient has separate column of adherence record for each follow up. So this method was used to summarize the adherence profile from multiple records of adherence variables. This is included in the method section now. |
| 4. | Line 156-158 Stepwise variable selection procedures, which include backward selection, usually don’t do a good job finding the most appropriate model.  Stepwise and any p-value based selections have a quite bit of evidence suggesting that they are poor at selecting appropriate variables. It’s better to select on more robust criteria, especially which assesses the fit of the model or, better yet, a penalized estimator such as lasso or lars. | True. The method of fitting the model was done by checking log-likelihood ratio with a variable being included in the model if it resulted in an improvement in the model fit. This was unnoticed error of writing. We are thankful for the critical appraisal, and great evidence from the listed literatures. |
| 5. | Line 157-158 Usually stepwise variable selection procedures select based on the p-values of the predictor variable, which isn’t a measure of model fit. If you have used something other than the default, that is mentioned here. Regardless, I strongly recommend not using stepwise selection procedures. | See above |
| 6. | Table 1 Significance testing in these situations is generally frowned upon because non-significant p-value doesn’t indicate that the groups are the same. | This approach was informed by the fact that testing for differences between the two groups can uncover some hidden issues. For example, if included and excluded groups differ with respect to address that may have a number of implications.  This said, we are willing to delete the p value column if the reviewer deems it necessary. |
| 7. | Table 2 How were the CI calculated for these rates? I don’t believe I saw that in the statistical method section. | These CI were generated by STATA v 12 thru the tab of statistics > survival analysis > Summary statistics, tests and tables > Person-time, incidence rates and SMR after defining the data in time to event form. |
| 8. | Line 251 Although I know the phrase “independent predictors” is used quite a bit, I don’t understand the usage of “independent” in a multivariable model since the effect is dependent on other predictors in the model. I recommend not using that phrasing and something along the lines of “after controlling for other factors.” | See above….. Amendments have been made. |
| **Points raised or amendments requested by Reviewer #4** | | **Authors’ response** |
| 1. | Main overall comment: Reordering of the results. Present Table 3 first then place table 2 followed by the cox modeling. | Changes have been made |
|  | **Abstract** |  |
| 2. | Line 33: Prevalence of TF is provided. It would be useful to also quote here the duration of follow up e.g., “prevalence was 38.5% (95%CI XX-XX) over a median follow-up of xx (xx-xx) | Comment well taken and the additional information has been provided. |
| 3. | Line 42: missing word (seven in one hundred) | Corrected |
|  | **Introduction** |  |
| 4. | Line 48: UNAIDS estimates are provided from 2019 in the first paragraph. Data for 2021 have now been released- update the references. | Necessary amendments have been made. |
| 5. | Line 59: Number of PLWHA in 2019 was 14000 (not IS. As now in the past). This figure is also quoted with a % - presumably, this is the % of the total population? This should be stated clear. | This is now stated clearly.it is 0.36% from the total population of Eritrea. |
| 6. | Line 60: This sentence starts with proving information on children then quotes the number on ART, which appears to relate to the total population, including adults. This could be reordered as this is not immediately clear. | This has been now rectified. |
| 7. | “ranging”- should be “range” | It is now corrected. |
|  | **Methods and Materials** |  |
| 8. | Line 91: States 822 children <15 years were seen in the clinic (this is the total shown in the flow diagram). Line 100 states all individuals <18 were included. Is the criteria to be seen in the clinic being < 15year, but patients were included in the analysis as long as they initiated ART by 18 years? Or were they only followed up to 18 years? Please clarify why the age cut offs differ. | The necessary amendment have been made…..The cut-off used is less than 18 years as the clinic holds children till they are 18 years or older. Therefore, the criteria have been uniformly changes all over the document. |
| 9. | Line 102: How is it possible to have unknown duration of follow up, and why is this treated differently from being LTFU? If a patient was seen in clinic and followed for certain time, couldn’t they be censored at the last point they were known to be in follow up? | We agree with this analysis…  One of the limitations of retrospective study is missing information.  That said, we must note that some a small number of patients had very inconsistent contact with the clinics and missing information on a range of variables – periods of follow up interspersed with periods of LTFU.  For consistency, treated them differently from those who are known to be lost to follow-up.  As you mentioned those who are lost to follow-up could be censored according to their therapy outcome till the last point they were known to be in follow up. |
| 10. | Line 108: Were missed ART doses self-reported by patients? This should be stated. | The adherence assessment was made using pill-count in each visit. |
| 11. | Line 123: ‘Persistent’ should be defined e.g., does it mean 2 CD4+ below the specified levels over a certain time? When was the failure deemed to have occurred? At the time of the first measurement? | See above. |
| 12. | Line 128: “<10%” should be “5 to <10%”. I noted the previous reviewer also queried the inclusion of adherence in the model. It should be inserted as time update variable than just binary outcome of suboptimal adherence? | True. Adherence could be altered with follow-up. Adherence was categorized as maladherent or good adherent due the fact that each patient has separate column of adherence record for each follow up. So this method was used to summarize the adherence profile from multiple records of adherence variables. |
| 13. | Line 134: Height-for-age is missing a “-” | Inserted. |
| 14. | Line 153: year of cART start was included in the model and not listed here. | Its included now. |
| 15. | Line 155: cART change frequency – please specify what constitutes change. | Change constitutes changes of cART regimen among the first line options due a reason other than therapy failure e.g., due to toxicity, drug-drug interaction, among others. |
|  | **Results** |  |
| 16. | Line 169: Before comparing those included/excluded it would be useful to state in text that 97 were excluded due to…. | The information is incorporated. |
| 17. | Line 172: authors state the majority of those included were wasted etc compared to those excluded. I think this should be “fewer” of the participants included were stunted, wasted etc. Eg. Only 25.6% of those included had weight for age < -2 compared to 56.5% of those excluded. | Well noted and the sentence is modified. |
| 18. | Line 188: The median time to TF is quoted, presumably this is median time in those who failed? This should be specified to distinguish between other estimates of median time to TF estimated using KM later in the results. | Yes. This is clarified. |
| 19. | Line 194: table 2. Why do the number of events in table 2 differ from table 3? EG 156 males with TF in table 2 and 157 in table 3? | The tables have minute of difference due trying to incorporate those who did not have follow up duration but had known therapy outcome.  This means in the table of crude-incidence you would find a little lower number than the prevalence table. |
| 20. | Line 204: table 3 presents row percentages, for e.g., showing what proportion of pt with WHO mild, moderate, and advanced stage had TF. The texting starting line 204 describes characteristics of those who failed compared to those who didn’t. While in the end the differences are equivalent/follow on from each other, from the text, I would expect to see column percentages in table 3 so the trends described can easily be seen (or else the text should be amended to better reflect the table format) | The percentages have been changed to column for a better understanding. |
| 21. | Line 238: at first use should specify the “median” quoted is ‘median time to failure’. | This is now incorporated |
| 22. | Line 240: ‘mean’ is given for some years. Further in line 242 its referred to as ‘average’ time to failure. Consistent language should be used (and assume it should be median throughout?) | This is now made consistent throughout the text. |
|  | **Discussion** |  |
| 23. | Line 273: the prevalence’s quoted in other countries don’t read well, please review sentence structure | The structure has been made rectified. |
| 24. | Line 340: ‘delays in the drug switching’- where/how was this assessed? | This was calculated from the data as it includes information on the time of therapy failure and date of therapy switch. Even though extensive analysis has not been undertaken, the general observation is that there is a delay between onset of TF and switch. |
| **Points raised or amendments requested by Reviewer #5** | | **Authors’ response** |
| 25. | Several minor stylistic or language corrections are required. | Extensive review of the documents has been undertaken to improve sentence structure and style. |
