# Supplementary material for: Prevalence and factors associated with pediatric HIV therapy failure in a tertiary hospital in Asmara, Eritrea: A 15-year retrospective cohort study
Source: PLoS One. 2023 Mar 9;18(3):e0282642. doi: 10.1371/journal.pone.0282642 (PMC9997912; doi:10.1371/journal.pone.0282642)
Supplement: Attachment — Submitted filename: Point by point response third round.docx [file pone.0282642.s004.docx]

**To PLOS ONE Editorial Office**

We would like to thank staff Editor of PLOS ONE and our reviewers for their invaluable inputs and constructive comments that are helpful to massively improve the quality of the manuscript. After careful consideration of the points raised, the point-by-point response to the third round of revision are as follows:

| **Minor Points raised or amendments requested by Academic Editor (Dr. CM González-Domenech)** | | | **Authors’ Response** |
| --- | --- | --- | --- |
| 1. | Comment 7 from Reviewer 3 related to CI calculation is not included in the Method section yet | | These CI were generated by STATA v 12 thru the tab of statistics > survival analysis > Summary statistics, tests and tables > Person-time, incidence rates and SMR after defining the data in time to event form.  Generally, these are equivalent to unadjusted HR with CI calculated in regard to their respective SE.  This statement has been incorporated in the third round of revision. |
| 2. | Comments 2 and 19 from Reviewer 4 were answered but not included in the corresponding sections, Method and Discussion, respectively | | Will be addressed |
| 3. | Line 100 of reviewed version: an “and” is missing (between duration, “and” unknown); line 226: One of the p for the p-values is differently in capital letter. | | The needed amendments have been carried out. |
| 4. | If applicable, we recommend that you deposit or indicate how the raw data could be accessed, as the reviewer 4 pointed. | | The raw data has been depositied along with reseach materials |
| #**Reviewer 4** | | | **Authors’ Response** |
| **Abstract** | | |  |
| 1. | Line 22 - Typo in first line – (should be “treatment failure IN”) | | Addressed |
| 2. | Line 29 – Data are plural, so should be “Data were” | | Addressed |
| 3. | Line 41 – score is missing “weight for height z-score”. AHR has a capital A, previously was aHR | | Addressed |
| 4. | Line 45 – The authors state seven in one hundred children likely to develop TF. The results is per 100 person years – so the conclusion should clarify its each year. | | Addressed |
| **Introduction** | | |  |
| 1. | Line 54 – what year does the 53% refer to? Reference is recent UNAIDS report but previous sentence refers to 2018 so this isn't clear. And is the 53% in the focus countries, or globally? | | The statement has been overhauled. The 53% represents the number of children covered by cART in focus countries. |
| **Methods** | | |  |
| 1. | Line 99 – the authors previously elaborated on what was meant by unknown follow up duration. However, in the manuscript I think this might still be unclear to the reader (“Additional exclusion considerations included unknown follow up duration, unknown therapy outcome endpoints (Fig 1)”. The figure describes this as “Key missing follow up data”. Should this be “Missing key follow up data”? Could you simply state in the manuscript – Unknown treatment outcome? Or even “missing key follow up data”? The issue is you don’t have the data to determine if/when the outcome occurred, but this isn’t really clear. | | Statement has been incorporated to make it clear |
| 2. | Line 120 – “6 months of effective treatment” – what is meant by effective? Or do you just mean after 6 months of being on treatment (as for the immunological definition). | | The word “effective” is erased as it’s a deplicate information as outcome with adherence support was already mentioned in line 117. |
| 3. | Line 124 – does the 3 months refer to time on treatment, or the space between VLs (could be clarified in text). | | overhauled |
| **Results** | | |  |
| 1. | Line 184 – p<0.03 – should be p=0.03 | | overhauled |
| 2. | Table 1: should p for weight for age be p<0.005? | | overhauled |
| 3. | Table 2 and 3 – the table numbers are the wrong way round in the table titles. | | overhauled |
| 4. | Line 213 – table 2 is referred to after the median time to cART switch – but that is not the information presented in the table. Table 2 should be properly introduced in the text. Eg “Crude incidence rates by key characteristics are provided in table 2” | | True. Changes has been made |
| 5. | Table 4: The authors explained in their response what was meant by cART change frequency, but I think would be helpful to include this in the manuscript – e.g. in the table footnote. | | Added |
| **Discussion** | | |  |
| 1. | Paragraph 1 – some studies give a time frame for duration of follow up – is it possible to say anything about the others? It’s difficult to interpret rates without information on time point/duration | | True, as the results are from different studies of different methodology, duration of follow-up couldn’t be obtained from all of them. Some are only cross-sectional, evaluating prevalence of TF while others are cohorts, evaluating both prevalence and incidences. |
| **Reviewer #3** | | | **Authors’ Response** |
| 1. | | (comment 6) Yes, I recommend removing the p-value column from table 1. | Its removed. |
| 2. | | (comment 7) I'm not familiar enough with Stata to know how this is calculated. If possible, I recommend including a methodological citation for how the CIs were calculated. This will depend on whether the Stata help has a citation. | It is now cited. |
