# Supplementary material for: Prevalence and factors associated with pediatric HIV therapy failure in a tertiary hospital in Asmara, Eritrea: A 15-year retrospective cohort study
Source: PLoS One. 2023 Mar 9;18(3):e0282642. doi: 10.1371/journal.pone.0282642 (PMC9997912; doi:10.1371/journal.pone.0282642)
Supplement: Attachment — Submitted filename: Response to reviewers.docx [file pone.0282642.s005.docx]

**To PLOS ONE Editorial Office**

We would like to thank staff Editor of PLOS ONE and our reviewers for their invaluable inputs and constructive comments that are helpful to massively improve the quality of the manuscript. After careful consideration of the points raised, the point-by-point response to the fourth round of revision are as follows:

1. Cite 23 is misspelled: Addressed.
2. P-values in table 2 and 3 should be in low case: The letters are changed to low case now.
3. Line 40: AHR, ‘A’ still in capital letter: The term adjusted hazards ratio has been abbreviated as aHR consistently throughout the document now.
4. Table 1. The label “Excluded” has been removed but the corresponding data still exists: The table is checked for any missing labels of the column, excluded but it still holds.
